# The Camden and Islington Viral Hepatitis Identification Tool (CIVHIT): Use of a Clinical Database Case‐Finding Tool for Hepatitis B, Hepatitis C and HIV in Primary Care

**DOI:** 10.1111/jvh.14027

**Published:** 2024-10-24

**Authors:** David Etoori, Sara Cococcia, Ankur Srivastava, Stuart Flanagan, Grainne Nixon, Satya Bobba, Alex Warner, Karen Sennett, Caroline Sabin, Sarah Morgan, William M. Rosenberg

**Affiliations:** ^1^ National Institute for Health and Care Research (NIHR) Health Protection Research Unit (HPRU) in Blood‐Borne and Sexually Transmitted Infections at UCL in Partnership With the UK Health Security Agency (UKHSA), Royal Free Campus London UK; ^2^ Division of Medicine & Royal Free London NHS Foundation Trust, Institute for Liver and Digestive Health University College London London UK; ^3^ Gastroenterology and Endoscopy Unit Cardinal Massaia Hospital Asti Italy; ^4^ Central and North‐West London NHS Foundation Trust London UK; ^5^ North‐East & Central London Health Protection Team, Public Health England London UK; ^6^ North Central London Integrated Care System, Laylock PDC London UK; ^7^ Caversham Group Practice London UK; ^8^ Medical Centre London UK; ^9^ Hampstead Group Practice London UK

**Keywords:** HBV, HCV, screening, viral hepatitis

## Abstract

Despite the availability of effective treatment and vaccines for hepatitis B virus (HBV) and C virus (HCV), many people are still infected and remain unaware of their infection. The Camden and Islington Viral Hepatitis Identification Tool (CIVHIT), a computer‐based search tool, was introduced in 60 general practices (GPs) in April 2014 to support identification, testing and treatment of individuals at high risk for blood‐borne viruses (BBVs). CIVHIT searched electronic medical records (EMRs), flagging all those with codes linked to risk factors or medical conditions associated with BBVs. CIVHIT was associated with a 78.5% increase in BBV tests in primary care in both boroughs. This translated to a 55.8% rise in new diagnoses. HBV testing saw the largest increase resulting in twice as many people diagnosed. Only 23.2% of HBV and 14.9% of HCV‐positive tests were referred to secondary care. In an index practice, the most common flag was a history of STIs (477/719, 66.3%). Individuals with previous or current drug use and those with a known hepatitis contact were more likely to be offered a test compared to those flagged due to a history of STI. HIV and HBV testing was lower in males following a test offer. There was an increased likelihood of testing for HBV and HCV with increasing age. Additionally, individuals with previous or current drug use and individuals with a known hepatitis contact were more likely to test for HCV compared to individuals flagged due to STI history. CIVHIT shows promise to assist with the elimination of BBVs.

**Abbreviations**
BBV: blood‐borne virus
CHB: chronic hepatitis B
CHC: chronic hepatitis C
DAA: directly acting antivirals
EMR: electronic medical record
GP: general practice
HBV: hepatitis B virus
HCC: hepatocellular carcinoma
HCV: hepatitis C virus
PWID: people who inject drugs

## 1 Introduction

Chronic viral hepatitis, including hepatitis B virus (HBV) and hepatitis C virus (HCV) infections, increases the risk of developing liver cirrhosis (up to 50%), liver cancer (1%–5%), and consequently death over three decades [1, 2]. Although several measures have led to a reduction in the incidence of viral hepatitis, including screening of the blood supply, reduction in the use of injected drugs, and the worldwide introduction of HBV vaccination [3], HCV and HBV continue to infect at least 1% and 3.5% of the global population, respectively [4]. The introduction of oral direct‐acting antiviral agents (DAAs) has created the possibility of curing over 95% of people infected with HCV [5]. Consequently, the World Health Organisation set the goal of eliminating HCV as a public health threat by 2030, and NHS England aims to achieve this goal by 2025 [6, 7].

To achieve elimination, an important first step is offering appropriate viral hepatitis testing to higher prevalence populations. In England, HCV testing principally occurs in primary care, where it accounts for as much as 30% of all HCV antibody (HCV Ab) positive tests [8]. Several studies have evaluated different approaches to increase testing in primary care. In the United States, the Hepatitis C Assessment and Testing Project (HepCAT) employed both birth‐cohort testing and risk‐based screening which led to increased case detection [9]. In risk‐based screening, 27.8% of those screened had at least one risk factor and seven factors identified all infections: intranasal or injected drug use, > 20 lifetime sexual partners, elevated ALT, transfusion before 1992, maternal HCV infection and existing liver disease [10]. In England, the Hepatitis C Assessment Treatment Trial (HepCATT) evaluated the impact of a primary care computer software program to help general practitioners (GPs) to identify people at higher risk. The software was used pro‐actively to generate a list of GP attendees at ‘high risk’, who were subsequently contacted and tested, according to predefined clinical information codes in their medical records. HepCATT reported an increase in the number of tests performed with a low increase in the HCV test yield [11].

All these interventions only addressed HCV infection. However, HBV and HCV infections share some risk factors, and many guidelines recommend simultaneous testing for HCV, HBV, and human immunodeficiency virus (HIV) in individuals thought to be at risk of any one of these blood‐borne viruses (BBVs) [12]. Furthermore, in areas of relatively high prevalence, testing for these three viruses is highly cost‐effective [13].

We aimed to improve case finding and facilitate referral and treatment of people infected with HCV, HBV or HIV by developing a computer‐based screening tool to identify at‐risk individuals in general practices in the London Boroughs of Camden and Islington. We report on the impact of the Camden and Islington Viral Hepatitis Identification Tool (CIVHIT) on testing and referrals to secondary care of people infected with BBVs in these boroughs. We also report on risk factors that prompted a CIVHIT flag and factors associated with the offer and uptake of testing in an index practice in the study area.

## 2 Methods

### 2.1 Intervention

The intervention had three components. First, there was an education component for GPs in the study areas (London boroughs of Camden and Islington), and second, a computer‐based risk flagging tool was introduced in all GPs (over 60) in the study area. Finally, this was supplemented with screening of all new registrants in GPs in the study areas.

#### 2.1.1 Educational Intervention

Before CIVHIT was introduced, all GPs working in the two selected London boroughs were offered an educational presentation on viral hepatitis, with a specific focus on risk factors and case‐finding strategies and on how to use CIVHIT. All participating GPs were encouraged to test all people registering at their practice for the first time, and all existing patients opportunistically identified by CIVHIT when their electronic medical record (EMR) was opened. GPs were at liberty to ignore or disable CIVHIT, and it was decided to not evaluate uptake of the programme for reasons of confidentiality.

#### 2.1.2 CIVHIT

CIVHIT was introduced in all general practices in the London boroughs of Camden and Islington in April 2014. CIVHIT is a computer‐based tool that searched the primary care Egton Medical Information System (EMIS) clinical information codes of all registered patients to identify codes linked to risk factors or medical conditions associated with HBV and HCV infections. Individuals were considered at high risk if they belonged to one of the following groups: (i) people born in a country with an estimated hepatitis B prevalence of 8% or more; (ii) people with a recorded family member with hepatitis B or C; (iii) men that have sex with men; (iv) commercial sex workers; (v) people with a history of a sexually transmitted infection; (vi) people who inject drugs (PWIDs); (vii) people on methadone medication; (viii) people with a previous transfusion; and (ix) people with a diagnosis of hepatitis B or C only but not both (Appendix S1). Each group was identified from records by a series of clinical information codes that would trigger the CIVHIT response and flag the relevant individuals in the GP EMR. A broad spectrum of clinical information codes was included to increase the sensitivity of the tool to identify people living with BBVs (Appendix S1). CIVHIT was installed onto each practice's clinical system by the practice manager. CIVHIT ran through each individual record until it identified the first of a series of Read codes associated with one of the prespecified risk factors associated with HCV, HBV or HIV (Appendix S1). Once a risk factor was triggered, the individual's record was flagged and the risk factor initiating the trigger was recorded. CIVHIT could be used in an ‘opportunistic’ mode or in a ‘target mode’. When run in the opportunistic mode, CIVHIT generated an alert in the form of a ‘pop‐up’ that appeared on the GP's desktop computer alerting them to ‘consider for Hepatitis screening’ every time they accessed the records of a person identified as being at‐risk. The triggering risk factor was not displayed but could be searched for. On the basis of their clinical judgement, GPs could then decide whether or not to act on the pop‐up. Alternatively, if run in ‘target mode’ and used pro‐actively, CIVHIT generated a report listing people deemed to be at risk and subdivided them into those known to have been tested and those with no record of testing.

#### 2.1.3 New Registrants at General Practices in Camden and Islington

New registrants were asked to complete a questionnaire at registration in accordance with routine practice. As part of the CIVHIT intervention, this questionnaire was adapted to incorporate questions relevant to BBV risk factors (Appendix S2). GPs were provided an algorithm to facilitate the opportunistic testing of people at high risk (Appendix S3). Additionally, when the answers to the questionnaire were uploaded onto EMIS Web, any answers corresponding to codes in the CIVHIT list were flagged in the individual's EMR triggering pop‐ups when GPs reviewed their EMRs at the time of their consultation. Although the pop‐up stated, ‘consider for Hepatitis screening’, GPs were encouraged to offer all individuals testing for HCV antibodies (HCV Ab), HBV surface antigen (HBsAg) and HIV Ab.

#### 2.1.4 Testing and Treatment

All those patients who tested HCV Ab positive were subsequently tested for HCV RNA. At discretion of the GP, patients could also be tested for Hepatitis B core Antibody IgG (HBcAb) and Hepatitis B surface Antibody (HBsAb) as part of the basic screening. GPs were encouraged to refer all people testing positive for HCV RNA or HBsAg for a consultation with a liver specialist who would further assess them for treatment depending on disease severity, comorbidities and patient preference. All individuals who tested positive for HIV and were not yet on treatment were referred to specialist services.

### 2.2 Evaluation

The CIVHIT evaluation involved two phases. First, an analysis of anonymised aggregate testing data from both participating boroughs and an in‐depth audit of CIVHIT use in an index practice.

#### 2.2.1 Phase 1—Evaluation of Opportunistic Use of CIVHIT

CIVHIT was uploaded and available to GPs on the computers of all practices in Camden and Islington from April 2014. The number of tests requested, rates of positivity and numbers of people referred to secondary care were gathered anonymously for 1 year before (April 2013–March 2014) and 1 year after (April 2014–March 2015) the introduction of the CIVHIT. Demographic characteristics and reason for testing were collected for those patients seen in secondary care.

#### 2.2.2 Phase 2—Index Practice Evaluation Exercise

To evaluate the performance of CIVHIT in more detail, a well‐engaged index practice was identified from those GP practices participating in the evaluation and CIVHIT was run in ‘target mode’ to generate a list of all those people identified as being at‐risk, regardless of prior testing. This phase started immediately following Phase 1 and run until 2019. Medical records were reviewed, and basic demographic data were collected as well as the reason for being flagged, the tests offered/declined/performed and all test results.

### 2.3 Statistical Analyses

Continuous data were described with mean and standard deviation (SD) or median and interquartile range (IQR) and categorical data as counts and percentages. The number of tests performed before and after the introduction of the CIVHIT was compared applying the Chi‐square test. A two‐sided *p* < 0.05 was considered statistically significant.

We determined factors associated with being offered a test following a CIVHIT flag, and factors associated with testing following a CIVHIT flag and test offer using logistic regression. Univariable analyses were run with each variable that could plausibly affect the outcomes with variables with *p* < 0.1 included in the multivariable analysis. The model of best fit was determined using Wald tests.

Analyses were performed in SPSS (Version 26.0. Armonk, NY: IBM Corp) and Stata 16 (College Station, TX: StataCorp LLC; 2017).

### 2.4 Governance and Ethics

The evaluation of the CIVHIT project was overseen by the Leads of the Camden and Islington Clinical Commissioning Groups (CCGs) (SM and KS) and their respective Boards and a consultant hepatologist (WMR). This service evaluation was registered on IRAS (RFHBU_77823/24) and reviewed by the Royal Free R&D Department and deemed to not require external ethical review as no identifiable data were analysed or included in the evaluation and only aggregate data were recorded and analysed. The in‐depth evaluation of the impact of CIHVIT in the index practice was performed by individuals employed in the practice as an evaluation of clinical practice, and only anonymised data were released to the research team.

## 3 Results

### 3.1 Overall Included Population

The introduction of CIVHIT was associated with a significant increase in the overall number of viral hepatitis screening tests performed in primary care, rising by 78.5% (from 8520 to 15,210, *p* < 0.001). This increase in testing also translated into a 55.8% increase in the number of new diagnoses (from 199 in 2013–2014 to 310 in 2014–2015, *p* < 0.001). Although the number of referrals to secondary care more than doubled (from 26 to 60) after the introduction of CIVHIT, this represented a 6.3% increase from 13.1% to 19.4% when expressed as a percentage of those newly diagnosed. The overall test positivity rate decreased by 0.30% after the introduction of CIVHIT. Over the study period, HBV testing increased most after the introduction of CIVHIT with the number of tests more than doubling (from 4286 to 8877, *p* < 0.001). As a consequence, twice as many people were diagnosed as HBV positive when compared to the previous year (increase from 68 to 142, *p* < 0.001). Of note, the rate of HBV positivity among those tested did not change after the introduction of CIVHIT, remaining at approximately 1.60%. In contrast, HCV testing did not increase as much as HBV testing following the introduction of CIVHIT with the number of HCV tests rising by 49.6% (from 4234 to 6333, *p* < 0.001). This yielded a 28.2% increase in new diagnoses of HCV (from 131 to 168, *p* < 0.001), but a drop in HCV test positivity from 3.09% to 2.65% (Table 1). Of those found to be either HBV or HCV positive, only 23.2% and 14.9%, respectively, were referred to secondary care.

**TABLE 1:** Number of tests and outcomes pre and post the introduction of the CIVHIT.

Year	HBV			HCV			All			Borough BBV statistics	
	Total tested	Positive	% Positive	Total tested	Positive	% Positive	Individuals tested	Positive	% Positive	Total pop*	Prevalence
Camden											
Pre: 2013–2014	1326	33	2.49	1564	58	3.71	2890	91	3.15	262,203	0.03
Post: 2014–2015	4415	79	5.96	2116	67	3.17	6531	146	2.24	260,260	0.06
2015–2016 (projected)	2284	68	5.13	2168	54	2.49	4452	122	2.74		
Islington											
Pre: 2013–2014	2960	35	1.18	2670	73	2.73	5630	108	1.92	229,441	0.05
Post: 2014–2015	4462	63	1.41	4217	101	2.40	8679	164	1.89	232,682	0.07
2015–2016 (projected)	4306	38	0.88	4306	136	3.16	8612	174	2.02		
Total											
Pre: 2013–2014	4286	68	1.59	4234	131	3.09	8520	199	2.34	491,644	0.04
Post: 2014–2015	8877	142	1.60	6333	168	2.65	15,210	310	2.04	492,942	0.06
2015–2016 (projected)	6590	106	1.61	6474	190	2.93	13,064	296	2.27		

* Total population evaluated in April 2014 and April 2015.

### 3.2 Index Practice Exercise

#### 3.2.1 Testing and Treatment Cascades

**FIGURE 1:**
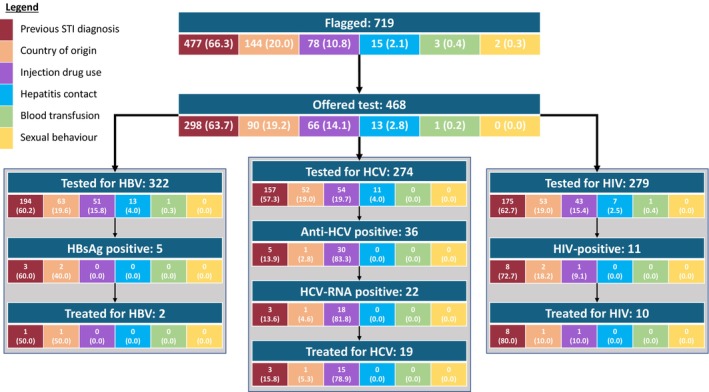
Flow of the patients flagged by the CIVHIT tool at the analysed index practice, including tests performed and outcomes. Two HBsAg‐positive patients did not need treatment.

A cohort of 15,843 people registered at the index practice was processed using CIVHIT, and 719 (4.8%) were flagged as ‘at risk’. In order of prevalence, the risk factor that triggered the CIVHIT flag was a previous diagnosis of a sexually transmitted disease (477/719, 66.3%), a high prevalence country of origin (144/719, 20.0%), current/previous drug use (78/719, 10.8%), contact with a person with HCV/HBV infection (15/719, 2.1%), prior blood transfusion (3/719, 0.4%) and sexual behaviour (e.g., sex work) (2/719, 0.3%) (Figure 1).

**FIGURE 2:**
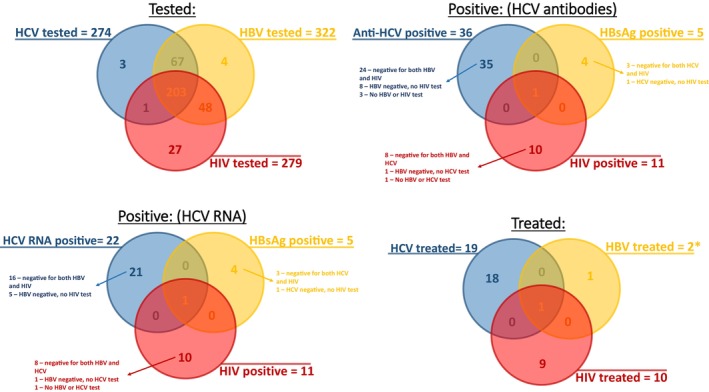
Overlap in testing, positivity and treatment uptake. *Of five HBsAG‐positive patients, two were treated, two did not require treatment and one was not treated.

Of the 719 people identified as being at risk of BBV by CIVHIT, testing for at least one of HBV, HCV and HIV was undertaken in 268 (37.3%) cases in primary care and 85 (11.9%) in secondary care. Of the 366 people who were not tested, 218 (59.6%) were never offered testing, 144 (39.4%) declined the offer of testing at least once and 4 (1.1%) were referred to sexual health services directly without testing in primary care. Of those 85 people (11.9%) who were first tested in secondary care, 27 had declined testing previously offered in primary care. Contrary to guidance provided to GPs when CIVHIT was launched not all people identified as at risk with the tool were tested for all viral infections, with 322 people tested for HBV, but only 274 for HCV and 279 for HIV likely representing a missed opportunity to test some individuals at risk (Figure 2). Among women offered BBV testing during the evaluation period, testing was triggered by pregnancy as part of routine antenatal care for 13.4%. These cases were not included in the evaluation. Of those tested for HBV, 1.6% (5/322) were HBsAg positive, indicative of HBV infection; 56% (42/75) were HBsAb positive and 20.9% (31/148) were HBcAb positive. The positivity rate for HCV Ab among those tested was 13.1% (36/274) of whom 61.1% (22/36) were also HCV RNA positive, indicative of infection with HCV. Of those tested for HIV, 4% (11/279) were found to be HIV Ab positive. Of those people testing positive for any of the infections, 13.9% (5/36) were lost to follow‐up, while the remaining were followed up in secondary care. All the HCV patients seen in secondary care were successfully treated (Figures 1 and 2).

#### 3.2.2 Factors Associated With Being Offered a Test

Of 719 individuals flagged by CIVHIT, 468 (65.1%) were offered a test. Individuals flagged due to previous or current drug use [OR: 3.94 (95% CI: 2.04–7.63), *p* < 0.001] and for having a known hepatitis contact [OR: 5.02 (95% CI: 1.07–23.63), *p* = 0.041] were more likely to be offered a test compared to those flagged due to a STI diagnosis. Younger (< 25 years) and older individuals (45–54 and 65+) were less likely to be offered a test (Table 2).

**TABLE 2:** Factors associated with being offered a test following a CIVHIT flag.

	Flagged	Test offered	OR (95% CI)	*p*	aOR (95% CI)	*p*
	719	468				
	*n* (%)	*n* (%)				
Reason for flag					**(*n* = 717)**	
Previously diagnosed STI	477 (66.3)	298 (63.7)	Reference	__	Reference	__
Country of origin	144 (20.0)	90 (19.2)	1.00 (0.68, 1.47)	0.995	1.14 (0.77, 1.70)	0.513
Drug use	78 (10.9)	66 (14.1)	3.30 (1.74, 6.28)	< 0.001	3.94 (2.04, 7.63)	< 0.001
Known hepatitis contact	15 (2.1)	13 (2.8)	3.90 (0.87, 17.50)	0.075	5.02 (1.07, 23.63)	0.041
Blood transfusion	3 (0.4)	1 (0.2)	0.30 (0.02, 3.33)	0.327	0.30 (0.03, 3.44)	0.335
Sexual behaviour	2 (0.3)	0 (0.0)	__	__	__	__
Age						
< 25	48 (6.7)	26 (5.6)	0.47 (0.24, 0.91)	0.026	0.40 (0.20, 0.80)	0.009
25–34	155 (21.6)	111 (23.7)	Reference	__	Reference	__
35–44	186 (25.9)	129 (27.6)	0.90 (0.56, 1.43)	0.649	0.89 (0.55, 1.43)	0.628
45–54	154 (21.4)	95 (20.3)	0.64 (0.40, 1.03)	0.065	0.54 (0.33, 0.89)	0.015
55–64	91 (12.7)	59 (12.6)	0.73 (0.42, 1.27)	0.267	0.62 (0.35, 1.10)	0.104
65+	85 (11.8)	48 (10.3)	0.51 (0.30, 0.89)	0.018	0.44 (0.25, 0.77)	0.004
Sex						
Female	424 (59.0)	269 (57.5)	Reference	__	__	__
Male	295 (41.0)	199 (42.5)	1.19 (0.87, 1.63)	0.267	__	__

#### 3.2.3 Factors Associated With Accepting a Test Offer

Guidance issued to GPs recommended testing for all three BBVs. However, GPs were elected to test for specific BBVs in some cases. Of 468 individuals offered a test, 353 (75.4%) were tested for at least one BBV. Males were less likely to be tested for HIV or HBV following a test offer [HIV OR: 0.66 (95% CI: 0.45–0.96), *p* = 0.032; HBV OR: 0.52 (95% CI: 0.34–0.78), *p* = 0.002]. There was an increased likelihood of testing for HBV and HCV with increasing age. Additionally, individuals flagged due to previous or current drug use [OR: 2.50 (95% CI: 1.23–5.08), *p* = 0.011] and individuals with a known hepatitis contact [OR: 6.03 (95% CI: 1.27–28.63), *p* = 0.024] were more likely to test for HCV compared to individuals flagged due to a history of a STI (Table 3).

**TABLE 3:** Factors associated with testing following a test offer.

Total	Test offered	Tested for HIV			Tested for HBV			Tested for HCV		
	468	279	aOR (95% CI)	*p*	322	aOR (95% CI)	*p*	274	aOR (95% CI)	*p*
	*n* (%)	*n* (%)			*n* (%)			*n* (%)		
Reason for flag			**(*n* = 468)**			**(*n* = 468)**			**(*n* = 467)**	
Previously diagnosed STI	298 (63.7)	175 (62.7)	__	__	194 (60.3)	__	__	157 (57.3)	Reference	__
Country of origin	90 (19.2)	53 (19.0)	__	__	63 (19.6)	__	__	52 (19.0)	0.99 (0.59, 1.64)	0.957
Drug use	66 (14.1)	43 (15.4)	__	__	51 (15.8)	__	__	54 (19.7)	2.50 (1.23, 5.08)	0.011
Known hepatitis contact	13 (2.8)	7 (2.5)	__	__	13 (4.0)	__	__	11 (4.0)	6.03 (1.27, 28.63)	0.024
Blood transfusion	1 (0.2)	1 (0.4)	__	__	1 (0.3)	__	__	0 (0.0)	__	__
Age										
< 25	26 (5.6)	13 (4.7)	0.82 (0.35, 1.94)	0.651	16 (5.0)	1.28 (0.53, 3.10)	0.588	15 (5.5)	1.80 (0.72, 4.46)	0.206
25–34	111 (23.7)	61 (21.9)	Reference	__	62 (19.3)	Reference	__	43 (15.7)	Reference	__
35–44	129 (27.5)	85 (30.5)	1.60 (0.95, 2.70)	0.08	84 (26.1)	1.50 (0.88, 2.54)	0.133	65 (23.7)	1.6 (0.95, 2.69)	0.078
45–54	95 (20.3)	62 (22.2)	1.59 (0.90, 2.80)	0.11	72 (22.4)	2.64 (1.43, 4.86)	0.002	63 (23.0)	2.73 (1.51, 4.94)	0.001
55–64	59 (12.6)	34 (12.2)	1.17 (0.62, 2.22)	0.631	49 (15.2)	4.31 (1.96, 9.47)	< 0.001	49 (17.9)	7.63 (3.36, 17.34)	< 0.001
65+	48 (10.3)	24 (8.6)	0.88 (0.44, 1.74)	0.704	39 (12.1)	3.93 (1.71, 9.00)	0.001	39 (14.2)	6.27 (2.71, 14.51)	< 0.001
Sex										
Female	269 (57.5)	172 (61.6)	Reference	__	198 (61.5)	Reference	__	151 (55.1)	__	__
Male	199 (42.5)	107 (38.4)	0.66 (0.45, 0.96)	0.032	124 (38.5)	0.52 (0.34, 0.78)	0.002	123 (44.9)	__	__

## 4 Discussion

This study evaluating an EMR‐based BBV testing intervention in GP practices in two London boroughs found that the CIVHIT tool was associated with increased testing and diagnosis of BBVs. The number of tests performed almost doubled following the introduction of CIVHIT compared to the previous year with HBV testing increasing the most following its introduction. Further investigation at the index practice found that the main reason for CIVHIT flagging a record was history of a STI. The risk most frequently associated with a positive BBV test was current or previous drug use.

Attempts to use computer‐based strategies to identify at‐risk cases in primary care in a range of settings have been reported. The HepCATT trial conducted in GP practices in Southwest England included use of an HCV audit tool to flag patients at high risk, staff training and an awareness campaign within intervention practices. The intervention was associated with a modest increase in HCV testing in intervention practices and a three‐ to six‐fold increase in linkage to specialist care [14]. There was significant overlap in the wider groupings of risk categories and search codes for both algorithms, but differences in yield. These differences in yield are difficult to disentangle but likely represent current and historical differences in the geographical locations and their underlying populations (i.e., characteristics and risk profiles), inherent differences in the algorithms and their implementation, minute differences in the search codes and differences in the timing of the studies (2014 vs. 2016); for example, DAA early access programmes for people with severe disease were introduced in 2015 [15].

The HepFREE cluster randomised trial which targeted immigrants in GP practices in areas with a high density of migrants also showed that targeted screening in primary care is effective at improving screening for BBVs but only when doctors were incentivised and supported [16]. The HepFREE trial also found combined case finding for HBV and HCV to be an effective strategy.

A systematic review of the association between HBsAg seropositivity and a STI diagnosis found a significant association with current or past syphilis as well as with a past unspecified STI [17]. This finding was corroborated by an analysis of routine data from a nationally representative network of general practices across all regions of England [18].

A study in GP practices in Glasgow which offered testing to individuals with previous injection drug use showed that this targeted case finding increased testing, identified more positive tests and could act as a means of re‐engagement for previously diagnosed individuals who had yet to access care and treatment [19]. Finally, several studies have shown that risk‐based testing identifies more cases of viral hepatitis [20, 21, 22].

We showed that the CIVHIT tool which targeted a broad spectrum of risk factors was associated with increased testing and diagnosis of HBV, HCV and HIV infection in a highly diverse setting. Across both boroughs, BBV testing almost doubled following the introduction of the CIVHIT tool. Previous research shows that testing numbers increased nationally in the study period likely due to increasing awareness and national and subnational initiatives and interventions which aimed to increase identification of individuals with HCV infection in anticipation of DAA introduction [8]. Our study likely contributed to this increase, but without individually linked data, we can only speculate to what extent. Previous research has shown a testing gap in primary care for numerous reasons [23]. Given the drive to eliminate HCV in the United Kingdom by 2025 and worldwide by 2030 [6, 7], our study shows that proactive case finding in primary care through flagging risk factors in EMRs could be an effective method to increase testing and case identification. In turn, this should lead to treatment of BBVs that should reduce transmission and ultimately help with elimination goals. This will require investment in the development and dissemination of computer‐based tools such as CIVHIT and training GPs in their use and actions to be taken following a positive flag. The relatively low referral rates for people testing positive for BBV suggest a need for further training about the actions to be taken following a positive test. Given the timing of the study when DAAs were being introduced and only available to patients with severe disease through early access programmes, this could represent a conscious decision by clinicians to delay referrals in anticipation of DAAs becoming more widely available [8] or patients deferring treatment until DAAs became available, due to treatment perceptions and concerns about side effects [24, 25].

In England, GPs are not authorised to dispense treatment for HCV, HBV or HIV, and so all people testing positive for viral hepatitis or HIV can only access treatment if they are referred to secondary care [23]. However, we found that the referral rates to catchment area hospitals did not increase significantly following the introduction of CIVHIT despite increased diagnoses. Possible explanations are that patients declined referral, GPs did not follow guidance on referral, patients did not attend referral appointments or patients were referred to hospitals outside the catchment area. Unfortunately, the study design prevented record linkage that would reveal the outcomes for individually identified participants.

Investigation of the index practice showed the potential of the CIVHIT tool. In this practice, 4.8% of patients were flagged as at risk of BBV infection. Of those flagged, 50% were tested with 10% of those testing positive for at least one of the BBVs. Of those diagnosed with a BBV, 81% received treatment. Importantly, for HCV which can now be cured following the introduction of DAAs, 86% of patients who were HCV RNA positive were treated. In fact, contrary to the finding at the borough level, we found that all patients that tested positive for a BBV in the index practice were referred to secondary care and all those that were not lost to follow‐up were treated. This likely shows heterogeneity in implementation of the cascade of testing and linkage to treatment for the different practices across the two boroughs. The index practice studied was closely engaged in the CIVHIT project, so the outcomes are likely to reflect the close adherence to the CIVHIT protocol. It is likely that more work will be needed to ensure that similar outcomes are achieved across different practices. However, even in the index practice, there was a large drop off between patients flagged by CIVHIT and patients who were ultimately tested. Furthermore, for those that received testing, not everyone received all three BBV tests, potentially missing some cases. Qualitative studies are needed to investigate the sources and reasons for variation in the uptake, use and adherence to the CIVHIT protocol.

The injection drug use flag had the highest yield of positive HCV tests corresponding with other studies that have shown that this group is at the highest risk for BBVs [26, 27]. However, the most common flag in the index practice was a previous STI diagnosis. This is likely to be attributable to increased STI testing in GP practices across London following the roll‐out of the National Chlamydia Screening Programme [28]. This finding in CIVHIT supports targeting people with STI diagnoses but may also demonstrate the value of incentivising GPs to seek new cases. The yield from tests conducted in people flagged with STI diagnoses adds to the increasing evidence of the association between an STI diagnosis and viral hepatitis seropositivity and could have significant implications regarding targeted testing especially in sexual health clinics.

Previous research has shown that clinicians are not always able to quickly and reliably assess BBV risk using electronic records and sometimes forget to test patients with risk factors [29, 30, 31]. Our findings show that CIVHIT can automate this process and has demonstrated the ability to identify undiagnosed individuals. Furthermore, NHS England is looking to implement similar tools (HepCATT, Patient Search Identification (PSI) tool developed by MSD [32, 33]) as a means to increase detection of BBVs and to help with the drive towards elimination. Our findings suggest that CIVHIT could contribute to this venture especially given its broad spectrum of risk factors, its incorporation of three important BBVs, its easy implementation and use of existing computer platforms. However, the low referral rates may indicate human resource constraints, ineffective implementation, including training, or a lack of motivation at practices. As such, financial incentives for diagnosis and referral might have a significant impact on referral rates as these incentives have been shown to be effective in other studies [16, 34]. Additionally, feedback regarding effectiveness of the CIVHIT at the time of implementation might have encouraged ongoing engagement with the tool.

While not currently recommended in clinical guidelines, our findings suggest that testing for viral hepatitis in sexual health clinics could have a significant yield. As such, targeted interventions in this setting could be another method used to increase diagnosis of viral hepatitis in England. For example, public health stakeholders in the United Kingdom run recurring national sexual health screening programmes, and these could be modified to include screening for viral hepatitis.

It would be valuable to conduct qualitative research into the implementation of CIVHIT to understand clinicians' decision making regarding implementation of the tool, which tests were offered and why some people who tested positive were not referred to secondary care. Research with practice patients could also help to clarify why some patients did not attend secondary care or receive treatment after a positive test. Future research will also be needed to understand why we saw no increase in referral rates to catchment area hospitals during the intervention period despite increases in testing and diagnoses. Future research is also needed to understand clinicians' decision making on whom to offer a test and which tests to perform.

The study had several strengths. First, a large diverse population and over 60 GP practices in two diverse London boroughs implemented the CIVHIT tool. The intervention was run in two boroughs estimated to have a high prevalence of BBVs. Furthermore, the intervention addressed both HBV and HCV and also showed utility for identifying HIV infection. Finally, as the tool was implemented by GP practices rather than a dedicated study team, our findings represent what can be expected from this intervention in the ‘real‐world’.

Limitations include that we did not explore the acceptability and practicalities of running the intervention with practices and particularly with clinicians. Furthermore, consequent on the decision to operate the introduction of CIVHIT as a service innovation with evaluation as opposed to a research study with informed consent, it was not possible to link positive tests to individual patient records or to determine which test was triggered by CIVHIT. As a result, we can only infer an association rather than causation between the intervention and increased testing in the study area. The algorithm was limited by EMR data quality, and if some information was incomplete, then some high‐risk individuals might have been missed. The algorithm also flagged the first Read code identified, but it is conceivable that some individuals had multiple risk factors. We also were unable to determine why some patients were offered tests while others were not.

It is important to note that clinicians were free to turn off notifications or choose to ignore them. Some GPs reported that they switched off CIVHIT after a few months and some did so intermittently when they were busy. As such, we cannot describe the extent to which our findings represent the full extent of the population that might have been flagged by the tool and therefore tested for BBVs. Similar studies need to consider the reality that GPs are often overburdened and have competing priorities, and automated prompts might annoy rather than engage them. As such, other interventions like education (to increase knowledge, awareness and understanding) and incentivisation might have more impact [35]. General Data Protection Regulation (GDPR) only allowed for investigation of one index practice to understand granular detail of the intervention. Findings from this practice are likely to differ from other practices where the intervention was implemented, particularly as it was an enthusiastic adopter of CIVHIT. We also did not have the opportunity to ask patients about their experience of the intervention. Furthermore, due to data sharing restrictions, we were unable to determine why only a small proportion of patients testing positive were referred to secondary care. Data sharing restrictions also meant we could not investigate attrition due to patient‐level factors such as hospital access and mobility (e.g., if patients moved away at any stage of the intervention) or if patients preferred testing in a sexual health clinic for more discretion. Finally, these findings may not be generalisable to settings outside of London.

In conclusion, CIVHIT was associated with increases in testing, diagnosis and treatment of BBVs and shows promise as a means to assist with the elimination of BBVs. It is likely that implementation of tools such as CIVHIT will yield benefit if linked to training and incentivisation of GPs.

## Author Contributions

All authors participated in drafting of the manuscript or critical revision of the manuscript for important intellectual content and provided approval of the final submitted version. Individual contributions are as follows: S.C. and D.E. interpreted data and wrote the manuscript, D.E. performed statistical analyses, S.C. and A.S. locally collected data and drafted a preliminary version of the manuscript, and W.R. and C.S. made the final critical revision for important intellectual contents. All authors approved the final version of the paper.

## Conflicts of Interest

C.S. has received funding for membership of Advisory Boards, Data Safety and Monitoring Panels and for the preparation of educational materials from Gilead Sciences, ViiV Healthcare, Janssen‐Cilag and MSD. All other authors declare no competing risks.

## Supporting information


**Appendix S1.** Clinical information codes searched by CIVHIT on EMIS Web.

**Appendix S2.** Questionnaire for new registrants at general practices in Camden and Islington.

**Appendix S3.** Opportunistic hepatitis B and C testing pathway for people at increased risk of infection in primary care.

## Data Availability

The authors have nothing to report.
