# Supplementary material for: The Camden and Islington Viral Hepatitis Identification Tool (CIVHIT): Use of a Clinical Database Case‐Finding Tool for Hepatitis B, Hepatitis C and HIV in Primary Care
Source: J Viral Hepat. 2024 Oct 24;32(4):e14027. doi: 10.1111/jvh.14027 (PMC11883453; doi:10.1111/jvh.14027)
Supplement: Supplementary file 1 — Appendix S1. Clinical information codes searched by CIVHIT on EMIS Web. [file JVH-32-0-s001.docx]

**Appendix 1: Hepatitis and HIV screening read codes list**

**Colour index:**

|  | Information and guidance within the document |
| --- | --- |
|  | Read codes used by Islington LES |
|  | Read codes used by Camden LES |
|  | Region – WHO estimates >8% hepatitis B prevalence |
|  | Countries with high prevalence of HCV not already listed as high HBV prevalence |

**At Risk Group:**

**“At risk group” are those who belong to one of the following groups:**

**A. Patients with an estimated hepatitis B prevalence of 8% in their country of origin**

**B. A record of a family member with hepatitis B**

**C. Men that have sex with men**

**D. Commercial sex workers**

**E. History of sexually transmitted infection**

**F. PWID**

**G. On Methadone medication**

**H. Transfusion**

**I. Any hepatitis B diagnosis**

**J. Any hepatitis C diagnosis**

1. **Country of Birth –**

**Patients with country of birth recorded were flagged for hepatitis B and C if they were born in a country where the WHO estimates >8% hepatitis B prevalence**

**America Region**

13f0 - Born in Argentina

13f1 - Born in Belize

13f2 - Born in Bolivia

13f3 - Born in Brazil

13f4 - Born in British Guyana

13f5 - Born in Canada

13f6 - Born in Chile

13f7 - Born in Columbia

13f8 - Born in Costa Rica

13f9 - Born in Ecuador

13fA - Born in El Salvador

13fB - Born in Grenada

13fC - Born in Guatemala

13fD - Born in Guyana

13fE - Born in Honduras

13fF - Born in Mexico

13fG - Born in Nicaragua

13fH - Born in Panama

13fI - Born in Paraguay

13fJ - Born in Peru

13fK - Born in Suriname

13fL - Born in USA

13fM - Born in Uruguay

13fN - Born in Venezuela

**Africa Region**

13g0 - Born in Algeria

13g1 - Born in Angola

13g2 - Born in Benin

13g3 - Born in Botswana

13g4 - Born in Burkina Faso

13g5 - Born in Burundi

13g6 - Born in Cambodia

13g7 - Born in Cameroon

13g8 - Born in Cape Verde Islands

13g9 - Born in Central African Republic

13gA - Born in Chad

13gB - Born in Comoros Islands

13gC - Born in Congo

13gD - Born in Djibouti

13gE - Born in Egypt

13gF - Born in Equatorial Guinea

13gp - Born in Eritrea

13gG - Born in Ethiopia

13gH - Born in Gabon

13gI - Born in Gambia

13gJ - Born in Ghana

13gK - Born in Guinea Bissau

13gL - Born in Guinea Republic

13gM - Born in Ivory Coast

13gN - Born in Kenya

13gO - Born in Lesotho

13gP - Born in Liberia

13gQ - Born in Libya

13gR - Born in Madagascar

13gS - Born in Malawi

13gT - Born in Mauritania

13gU - Born in Mauritius

13gV - Born in Morocco

13gW - Born in Mozambique

13gX - Born in Namibia

13gY - Born in Niger

13gZ - Born in Nigeria

13ga - Born in Rwanda

13gb - Born in Sao Tome and Principe

13gc - Born in Senegal

13gd - Born in Sierra Leone

13ge - Born in Somalia

13gf - Born in South Africa

13gg - Born in Sudan

13gh - Born in Swaziland

13gi - Born in Tanzania

13gj - Born in The Gambia

13jB - Born in Togo

13gk - Born in Tunisia

13gl - Born in Uganda

13gm - Born in Zaire

13gn - Born in Zambia

13go - Born in Zimbabwe

**Asian Region**

13e0 - Born in Afghanistan

13e1 - Born in Armenia

13e2 - Born in Bahrain

13e3 - Born in Bangladesh

13e4 - Born in Bhutan

13e5 - Born in Brunei

13e6 - Born in Burma

13e7 - Born in Chechnya

13e8 - Born in China

13e9 - Born in Democratic People's Republic of Korea

13eA - Born in East Timor

13eB - Born in Georgia

13eC - Born in Hong Kong

13eD - Born in India

13eE - Born in Indonesia

13eF - Born in Iran

13eG - Born in Iraq

13eH - Born in Israel

13eI - Born in Japan

13eJ - Born in Jordan

13eK - Born in Kazakhstan

13eL - Born in Kuwait

13eM - Born in Kyrgyzstan

13eN - Born in Laos

13eO - Born in Lebanon

13eP - Born in Malaysia

13eQ - Born in Maldives

13eR - Born in Mali

13eS - Born in Mongolia

13eT - Born in Nepal

13eU - Born in North Korea

13eV - Born in Oman

13eW - Born in Pakistan

13eX - Born in Palestine

13eY - Born in Philippines

13eZ - Born in Qatar

13ea - Born in Republic of Korea

13eb - Born in Russia

13ec - Born in Saudi Arabia

13ed - Born in Singapore

13ee - Born in South Korea

13ef - Born in Sri Lanka

13eg - Born in Syria

13eh - Born in Taiwan

13ei - Born in Tajikistan

13ej - Born in Thailand

13ek - Born in Turkey

13el - Born in Turkmenistan

13em - Born in United Arab Emirates

13en - Born in Uzbekistan

13eo - Born in Vietnam

13ep - Born in Yemen

**Atlantic Region**

13j0 - Born in Antigua and Barbuda

13jE - Born in Aruba

13j1 - Born in Bahamas

13j2 - Born in Barbados

13j0 - Born in Antigua and Barbuda

13jE - Born in Aruba

13j1 - Born in Bahamas

13j2 - Born in Barbados

EMISNQBO20 - Born in Bermuda

13j3 - Born in Cuba

13j4 - Born in Dominican Republic

13j5 - Born in Haiti

13j6 - Born in Jamaica

EMISNQBO21 - Born in Martinique

EMISNQBO19 - Born in Montserrat

13j7 - Born in Puerto Rico

13j8 - Born in St. Kitts and Nevis

13j9 - Born in St. Lucia

13jA - Born in St. Vincent

13jC - Born in Trinidad and Tobago

**Australia Region**

13h0 - Born in Australia

13h1 - Born in New Zealand

**European Region**

13d0 - Born in Albania

13d1 - Born in Andorra

13d2 - Born in Austria

13d3 - Born in Azerbaijan

13d4 - Born in Belgium

13d5 - Born in Belorussia

13d6 - Born in Bosnia - Herzegovina

13d7 - Born in Bulgaria

13d8 - Born in Croatia

13d9 - Born in Cyprus

13dA - Born in Czech Republic

13dB - Born in Denmark

13dC - Born in England

13dD - Born in Estonia

13dE - Born in Finland

13dF - Born in France

13dG - Born in Germany

EMISNQBO22 - Born in Gibraltar

13dH - Born in Greece

13dI - Born in Hungary

13dJ - Born in Iceland

13dK - Born in Ireland

13dL - Born in Italy

13dM - Born in Kosovo

13dN - Born in Latvia

13dO - Born in Liechtenstein

13dP - Born in Lithuania

13dQ - Born in Luxembourg

EMISNQBO18 - Born in Macedonia

13dR - Born in Malta

13dS - Born in Moldavia

13dT - Born in Monaco

13do - Born in Montenegro

13dU - Born in Northern Ireland

13dV - Born in Norway

13dW - Born in Poland

13dX - Born in Portugal

13dY - Born in Republic of Ireland

13dZ - Born in Romania

13da - Born in San Marino

13db - Born in Scotland

13dc - Born in Slovakia

13dd - Born in Slovenia

13de - Born in Spain

13df - Born in Sweden

13dg - Born in Switzerland

13dh - Born in The Netherlands

13di - Born in Ukraine

13dj - Born in Vatican City

13dk - Born in Wales

13dl - Born in Yugoslavia

13dm - Born in former Yugoslav Republic of Macedonia

13dn - Born in Serbia

**Pacific region**

13k0 - Born in Fiji

13k1 - Born in Kiribati

13k2 - Born in Nauru

13k3 - Born in Papua New Guinea

13k4 - Born in Seychelles

13k5 - Born in Solomon Islands

13k6 - Born in Tonga

13k7 - Born in Tuvalu

13k8 - Born in Vanuatu

13k9 - Born in Western Samoa

1. **Family History of:**

FH: Infectious disease (123 - FH: Infectious disease)

1. **Men who have sex with men (MSM) –**

Gender- MALE

E220z - Homosexuality NOS

E2252 - Trans-sexuality with homosexual history

13m0 – Bisexual

1. **Sex workers –**

0AL - Sex worker

14Of - Former sex worker

0AK3 - Child prostitute

1. **History of sexual transmitted infection –**

**Codes for diagnosis of the following infections to be identified.**

**History of Chlamydia:**

14150 - H/O: chlamydia infection

43U8 - Chlamydia test positive

A78A0 - Chlamydial infection of lower genitourinary tract

A78A1 - Chlamydial infection of pharynx

A78A2 - Chlamydial infection of anus and rectum

A78A4 - Chlamydial conjunctivitis

A78AW - Chlamydial infection, unspecified

K40y1 - Female chlamydial pelvic inflammatory disease

A78A% Chlamydial infection

**History of Gonorrhoea**

14151 - H/O: gonorrhoea

K44 - Female gonococcal pelvic inflammatory disease

A980 - Acute gonorrhoea of lower genitourinary tract

A981 - Acute gonorrhoea of upper genitourinary tract

A982 - Chronic gon*orrhoea* lower genitourinary tract

A983 - Chronic gonorrhoea of upper genitourinary tract

A98z - Gonococcal infections NOS

4JQA / 4JQA - Gonorrhoea test positive

A98 - Gonococcal infections

**History of Syphilis**

A90 - Congenital syphilis

A93 - Cardiovascular syphilis

F4A54 - Keratitis due to syphilis

K0y0 - Late syphilis of kidney

1232 - FH: Syphilis

Ayu4A - [X]Syphilis, unspecified

K2142 - Prostatitis in syphilis

A910 - Primary genital syphilis

A911 - Primary anal syphilis

A97z - Syphilis NOS

K40y0 - Female syphilitic pelvic inflammatory disease

4382 - Syphilis titre test positive

**History of Genital herpes/ warts**

14152 - H/O: genital warts

A7812 - Genital warts

A7817 - Recurrent genital warts

14153 - H/O: genital herpes

A541 - Genital herpes simplex

A5410 - Genital herpes unspecified

A5411 - Herpetic vulvovaginitis

A5412 - Herpetic ulceration of vulva

A5413 - Herpetic infection of penis

A5415 - Anogenital herpes viral infection

A541z - Genital herpes simplex NOS

1. **People who inject drugs (PWID) –**

**Current and ex PWIDs can be identified by searching for read codes for injecting drug use AND methadone use**

**Drug User:**

13c0 / 13c0 - Injecting drug user

13c1 - Intravenous drug user

13cJ / 13cJ - Previously injecting drug user

1V30 - Injects drugs subcutaneously

1V31 - Injects drugs intramuscularly

**H/O Methadone misuse:**

1T10 - H/O daily methadone misuse

1T11 - H/O weekly methadone misuse

1T12 - H/O infrequent methadone misuse

1T13 - Previous history of methadone misuse

**Methadone Dependence:**

8B23-1 - Drug addiction therapy-methadone

8B2N / 8B2N - Drug addiction detoxification therapy - methadone

8B2P - Drug addiction maintenance therapy - methadone

8BE0 - Reinduction to methadone maintenance therapy

E240-2 - Methadone dependence

SL502 - Methadone poisoning

T801 - Accidental poisoning by methadone

TJ51 - Adverse reaction to methadone

U6050 - [X]Opioids + related analgesics cause adverse eff in therapy use

8B2R - Drug addiction detoxification therapy - buprenorphine

**Heroin misuse:**

1T0 - H/O heroin misuse

1T00 - H/O daily heroin misuse

1T01 - H/O weekly heroin misuse

1T02 - Previous history of heroin misuse

1T03 - H/O infrequent heroin misuse

1TE - Uses heroin on top of substitution therapy

Failed heroin detoxification (146C - Failed heroin detoxification)

**Heroin Dependence:**

Eu112 - [X]Mental and behavioural disorder due to use opioids: dependence syndrome

E240 - Opioid type drug dependence

SL501 - Heroin poisoning

T800 - Accidental poisoning by heroin

TJ50 - Adverse reaction to heroin, diamorphine

U1A5 - [X]Accident poisoning/exposure to narcotic drug

U205 - [X]Intent self poison/exposure to narcotic drug

1. **Transfusion**

Blood transfusion (7L143-1 - Blood transfusion) before 1991

H/O: blood transfusion (14S1 - H/O: blood transfusion)

1. **Hepatitis B Screening:**

9Op2 - Hepatitis B screening offered

8I3u - Hepatitis B screening declined

68280 - Hepatitis B screening required

43B - SH-antigen (hepatitis B) test

43B8 - Hepatitis B core antigen test

43B9 - Hepatitis B e antigen test

4JR1 - Hepatitis B screening test

6828/ 6828 - Hepatitis B screening

1. **Hepatitis B screening Result:**

9kZ - Hepatitis B screening positive - enhanced services admin

9kW - Hepatitis B screening negative - enhanced services admin

43d9 - Hepatitis B surface antigen level

43d8 - Hepatitis B surface antibody level

43dB - Hepatitis B core antibody level

43B2 - Hepatitis B immune

65W5 - Requires a course of hepatitis B

43B4 - Hepatitis B surface antig +ve

43B6 - Hepatitis B non-immune

43B9 - Hepatitis B e antigen test

43dC - Hepatitis B e antibody level

43dA - Hepatitis B core IgM level

43B8 - Hepatitis B core antigen test

65O3 - Anti-Hepatitis B immunoglob.

A7070 - Chronic viral hepatitis B with delta-agent

A7071 - Chronic viral hepatitis B without delta-agent

43BA - Hepatitis B surface antigen negative

1. **Hepatitis B Vaccination:**

65F1 - 1st hepatitis B vaccination

65F2 - 2nd hepatitis B vaccination

65F3 - 3rd hepatitis B vaccination

65F6 - 4th hepatitis B vaccination

65F7 - 5th hepatitis B vaccination

65FM - Sixth hepatitis B vaccination

65F4 - Boost hepatitis B vaccination

EMISNQ1S3 - 1st hepatitis B junior vaccination

EMISNQ2N3 - 2nd hepatitis B junior vaccination

EMISNQ3R2 - 3rd hepatitis B junior vaccination

EMISNQ4T1 - 4th hepatitis B junior vaccination

EMISNQBO14 - Booster hepatitis B junior vaccination

65MD - First combined hepatitis A and B vaccination

65ME - Second combined hepatitis A and B vaccination

65MF - Third combined hepatitis A and B vaccination

65MG - Booster combined hepatitis A and B vaccination

EMISNQBO7 - Booster combined paediatric hepatitis A and B vaccination

68Nm - No consent for hepatitis B vaccination

8I2e - Hepatitis B vaccination contraindicated

14b2 - History of three hepatitis B vaccinations

8I3r- Hepatitis B immunisation declined

68Nm - Hepatitis B immunisation refused

1. **Hepatitis B Diagnosis:**

ZV02B - [V]Hepatitis B carrier

A703 - Viral (serum) hepatitis B

A7073 - Chronic viral hepatitis B

A702 Viral hepatitis B with coma

A7070 Chronic viral hepatitis B with delta-agent

A7071 Viral hepatitis B without delta-agent

1. **Hepatitis C Screening:**

9Op1 - Hepatitis C screening offered

677Q - Hepatitis C screening counselling

8I3v - Hepatitis C screening declined

6829 / 6829 - Hepatitis C screening

1. **Hepatitis C screening Result:**

43X4 - Hepatitis C antibody test negative

43X3 - Hepatitis C antibody test positive

43X2 - Hepatitis C antibody test

43h3 - Hepatitis C PCR

2J1 - Hepatitis C status

43X6 - Hepatitis C antibody level

2J11- Hepatitis C immune

2J1 - Hepatitis C status

2J12 - Hepatitis C non immune

43B7 - Hepatitis C non-immune

43dD - Hepatitis C recombinant immunoblot assay

43j5 - Hepatitis C nucleic acid detection

43k1 - Hepatitis C antigen level

43q - Hepatitis C virus RNA assay

4J3B - Hepatitis C viral load

1. **Hepatitis C Diagnosis**

A70z0 / A70z0 - Hepatitis C

A7072 / A7072 - Chronic viral hepatitis C

ZV02C / ZV02C - [V]Hepatitis C carrier

**7Q053** – RSV treatment and hepatitis C treatment drugs band 1

65PM - Hepatitis C contact

65Q7 - Viral hepatitis carrier

677Q - Hepatitis C screening counselling

9kV - Hepatitis C screening positive - enhanced services admin

A7040 / A7040 - Viral hepatitis C with coma

A7050 /A7050 - Viral hepatitis C without mention of hepatic coma

1. **HIV Screening**

A788-1 - Human immunodeficiency virus infection

A789 - Human immunodeficiency virus resulting in other disease

43C2 - HTLV-3 antibody negative

43C3 - HTLV-3 antibody positive

AyuC5 - HIV resulting on other infectious and parasitic diseases

9Op0 - HIV screening offered

8I3p - HIV screening declined

1. **Referral**

8Hk5 - Referred to hepatology service

1. **Follow-up**

6828 - Hepatitis B screening

6829 - Hepatitis C screening

4JRF - Viral hepatitis screening test
