# Supplementary material for: The Camden and Islington Viral Hepatitis Identification Tool (CIVHIT): Use of a Clinical Database Case‐Finding Tool for Hepatitis B, Hepatitis C and HIV in Primary Care
Source: J Viral Hepat. 2024 Oct 24;32(4):e14027. doi: 10.1111/jvh.14027 (PMC11883453; doi:10.1111/jvh.14027)
Supplement: Supplementary file 2 — Appendix S2. Questionnaire for new registrants at general practices in Camden and Islington. [file JVH-32-0-s003.docx]

**History page:**

**Risk Criteria: Page 1**

**Risk Criteria: Page 2**
