# Supplementary material for: The Camden and Islington Viral Hepatitis Identification Tool (CIVHIT): Use of a Clinical Database Case‐Finding Tool for Hepatitis B, Hepatitis C and HIV in Primary Care
Source: J Viral Hepat. 2024 Oct 24;32(4):e14027. doi: 10.1111/jvh.14027 (PMC11883453; doi:10.1111/jvh.14027)
Supplement: Supplementary file 3 [file JVH-32-0-s002.docx]

Opportunistic hepatitis B and C testing pathway for people at increased risk of infection in primary care.

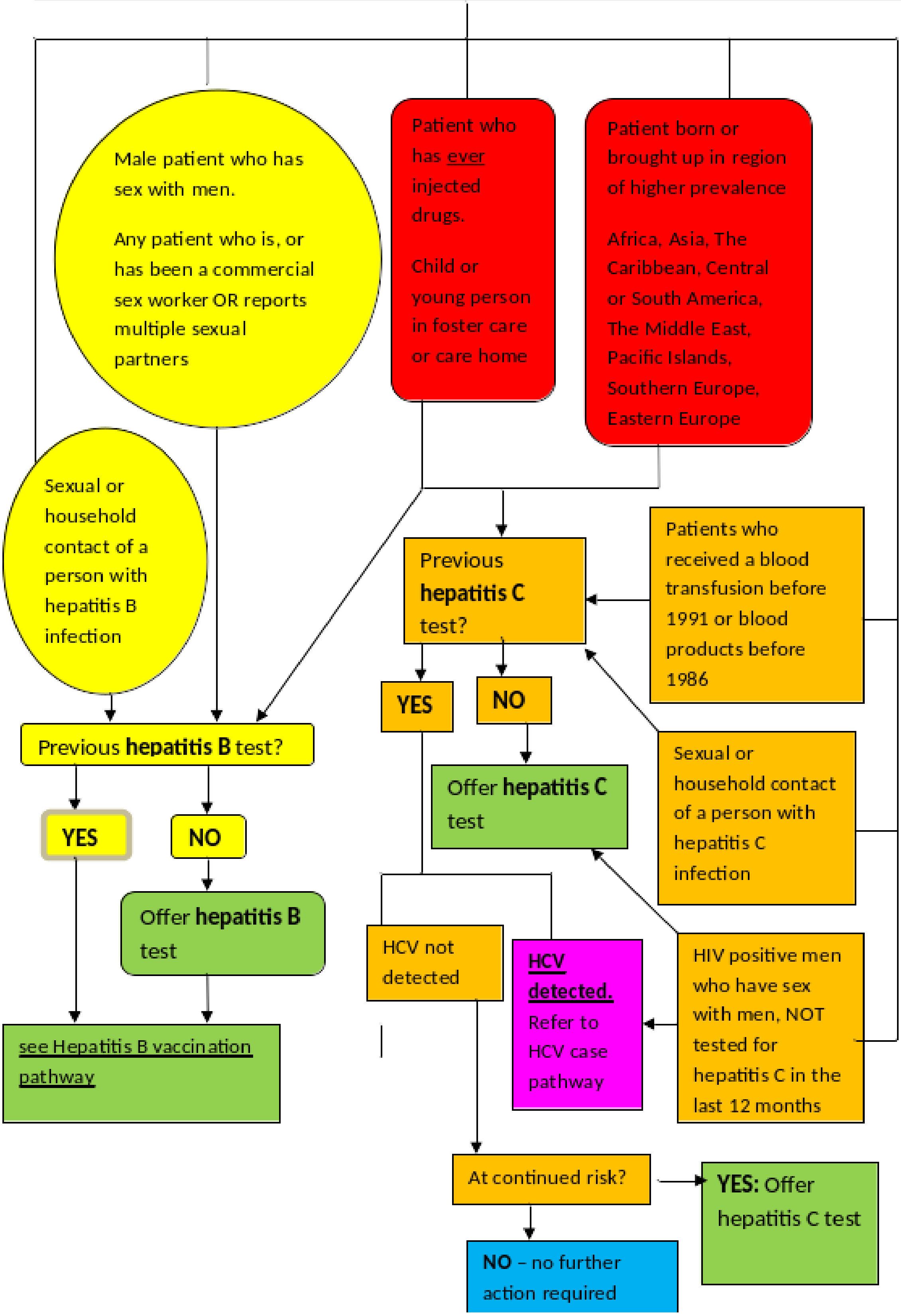

Hepatitis B vaccination pathway

Previous hepatitis B test?

NO → At continued risk?

YES →

- **HBsAg & anti-HBc <u>not</u> detected** → At continued risk?
- **HBsAg <u>detected</u>** Refer to HBV case pathway
- Anti-HBc detected. HBsAg not detected. **Patient immune NFA**

At continued risk?

Current intravenous drug user (also need hepatitis A vaccination).
Man who has sex with men (also need hepatitis A vaccination)
Patient who changes sexual partners frequently.
Household contact of a person who has chronic hepatitis B infection.
Foster carers (whole family).
Families adopting children from high prevalence region.
Patients who regularly receive blood or blood products **NB patients with haemophilia also require hepatitis A vaccination.**
Patients with chronic liver disease - including chronic hepatitis C infection (also need hepatitis A vaccination).
People residing in residential accommodation for people with learning difficulties.
People who regular travel to countries of higher prevalence (travel to Asia, Africa, south and central America also require hepatitis A vaccination.
People at occupational risk of hepatitis B and A see:
http://media.dh.gov.uk/network/211/files/2012/07/chap-18.pdf

http://media.dh.gov.uk/network/211/files/2012/07/chap-17.pdf

NO – no further action required

YES → **Test for hepatitis B if never tested or if >6 months since last test**

Hepatitis B risk only: offer monovalent hepatitis vaccination at 0, 1, 2 and 12 months

Vaccine	Ages	HBV Dose	Volume
Engerix B®	0–15 years*	10μg	0.5ml
Engerix B®	16 years or over	20μg	1.0ml
HBvaxPRO Paediatric®	0–15 years	5μg	0.5ml
HBvaxPRO®	16 years or over	10μg	1.0ml

See DH guidelines for dialysis patients http://immunisation.dh.gov.uk/green-book-chapters/chapter-18/

Hepatitis A and B risk – offer combined vaccination at 0.1.2 and 12 months

Vaccine	Ages	HAV dose	HBV dose	volume
Twinrix Adult®	16 years or over	720 ELISA units	20ìg	1.0ml
Twinrix Paediatric®	1–15 years	360 ELISA units	10ìg	0.5ml
